# Data on the relationship of emotional intelligence and stages of change among Malaysian prison inmates

**DOI:** 10.1016/j.dib.2021.106804

**Published:** 2021-01-29

**Authors:** Ida Hartina Ahmed Tharbe, Muhamad Khairul Anwar Kamaruddin, Melati Sumari, Ibrahim Maclean Chong

**Affiliations:** a Department of Educational Counseling, Faculty of Education, University Malaya, Kuala Lumpur, Malaysia; b Ministry of Home Affairs, Malaysia, Federal Territory of Putrajaya, Malaysia

**Keywords:** Emotional intelligence, Stages of change, Inmates, Prison, Rehabilitation

## Abstract

The data presented in this article examine the relationship between the subcomponents of emotional intelligence (emotional perception and expression, emotional facilitation of thinking, emotional understanding and emotional management) and the stages of change (pre-contemplation, contemplation, action and maintenance). The final data were obtained from 429 Malaysian inmates (374 male and 55 female) recruited from eight Malaysian prisons in four different zones. The two instruments used were the Self-Rated Malaysian Emotional Intelligence Scale (SRMEIS) and the University Rhodes Island Change Assessment Scale (URICA). Both instruments underwent expert validation through construct and test-retest validity. The researcher randomly distributed a total of 550 questionnaires, of which 429 were accepted and 121 were rejected due to missing data and outliers, resulting in 78% of participants providing data that could be used in the analyses. All participants were informed of the confidentiality of their data, and their participation was voluntary. SPSS and Excel files are provided as supplementary material.

## Specifications Table

**Table:** 

Subject	Psychology and counselling
Specific subject area	Emotional intelligence, stage of change, inmate rehabilitation.
Type of data	Table
How data were acquired	A survey carried out with questionnaires (See ‘questionnaire’ file in the supplementary file)
Data format	Raw and analysed data
Parameters for data collection	Inmates participated in the study on a volunteer basis. All participants were briefed about the confidentiality of participation before signing the informed consent form. Permission was also acquired from the Malaysian Prison Department.
Description of data collection	The data were collected manually between March 2020 and April 2020 from 550 inmates in eight Malaysian prisons. The authors administered data enumeration.
Data source location	Institution: Malaysian Prison Department Country: Malaysia
Data accessibility	http://dx.doi.org/10.17632/jw4829m2t6.3

## Value of the Data

• The data is valuable since research on inmates is still lacking everywhere in the world. Inmates are often regarded as a minority population with a different cultural complexity, and this data can assist in easing some of the misunderstanding towards this population.
• This data will be useful for Malaysian prison policy makers, rehabilitation and psychology officers in identifying the need for behavioural and emotional interventions for inmates, thus leading to the formulation of effective methods of intervention in prison rehabilitation services in Malaysia.
• The study adds to the body of knowledge needed for researchers interested in studying the relationship between socio demographics, emotional intelligence and stages of change among inmates.

## 1 Data Description

This paper presents the data obtained from questionnaires (see “questionnaire” file in the supplementary file) that measured the four dimensions of emotional intelligence (emotional perception, emotional facilitation of thinking, emotional understanding, emotional management) and the stages of change from the transtheoretical model (pre-contemplation, contemplation, action, and maintenance). The sample consists of 429 Malaysian inmates from eight prisons in Malaysia. The raw data shows the demographic profiles of the respondents (gender, age, religion, ethnicity, occupation, monthly income, level of education, and frequency of prison re-entry) in Table 1, and the data on correlation between emotional intelligence subscales and stage of change subscales in Table 2.

**Table 1:** Demographics profiles of sample in frequency and percentage.

Demographic		Frequency	Percentage
**Gender**	Male	374	87.2
	Female	55	12.8
**Age**	21–30 years old	183	42.7
	31–40 years old	141	32.9
	41–50 years old	75	17.5
	51 years old above	30	7.0
**Religion**	Muslim	289	67.4
	Buddhist	41	9.6
	Hindu	56	13.1
	Christian	42	9.8
	Others	1	0.2
**Ethnicity**	Malay	239	55.7
	Indian	64	14.9
	Chinese	56	13.1
	Others	70	16.3
**Occupational Affiliation**	Public Sectors	43	10.0
	Private Sectors	93	21.7
	Self Employed	260	60.6
	Student	33	7.7
**Monthly Income**	Below MYR 1000	187	43.6
	MYR 1001- MYR 3000	208	48.5
	Above MYR 3001	34	7.9
**Level of Education**	Primary School	53	12.4
	Secondary School	298	69.5
	Diploma	50	11.7
	Undergraduate	28	6.5
**Frequency of prison re-entry**	1st time	168	39.2
	2 - 5 times	194	45.2
	6 times and more	67	15.6

MYR = Malaysian Ringgit.

**Table 2:** Correlation between emotional intelligence subscales and stage of change subscales (see “data” file in supplementary file).

	Variables		Mean	SD	1	1a	1b	1c	1d	2	2a	2b	2c	2d
1	Emotional Intelligence		136.76	15.44	1	.756⁎⁎	.884⁎⁎	.248⁎⁎	.864⁎⁎	.326⁎⁎	.181⁎⁎	.410⁎⁎	.460⁎⁎	.372⁎⁎
1a	Emotional Perception And Expression		38.26	5.62		1	.469⁎⁎	.019	.515⁎⁎	.340⁎⁎	.173⁎⁎	.206⁎⁎	.319⁎⁎	.189⁎⁎
1b	Emotional Facilitation of Thinking		47.29	6.77			1	.120*	.755⁎⁎	.458⁎⁎	.028	.464⁎⁎	.462⁎⁎	.363⁎⁎
1c	Emotional understanding		12.29	2.58				1	.080	.209⁎⁎	.309⁎⁎	−0.009	.025	.186⁎⁎
1d	Emotional Management		35.32	4.74					1	.456⁎⁎	.166⁎⁎	.384⁎⁎	.389⁎⁎	.330⁎⁎
2	Readiness to Change		8.44	1.86						1	−0.529⁎⁎	.854⁎⁎	.844⁎⁎	.627⁎⁎
2a	Pre-Contemplation		2.88	.83							1	−0.170⁎⁎	−0.180⁎⁎	.118*
2b	Contemplation		3.88	.64								1	.779⁎⁎	.579⁎⁎
2c	Action		3.84	.62									1	.536⁎⁎
2d	Maintenance Striving		3.59	.56										1

⁎⁎ Correlation is significant at the 0.01 level (2-tailed).

⁎ Correlation is significant at the 0.05 level (2-tailed).

The respondents of this study consisted of 374 male and 55 female inmates from eight prisons in Malaysia. The male inmates represented 87.2% of the respondents. According to the data in World Prison Brief [1], female prisoners only constitute 4.5% of the prison population in Malaysia. Therefore, the imbalance in gender representation in the current study is expected. In terms of age range, 42.7% were aged between 21 and 30 years old, 32.9% between 31 and 40 years old, 17.5% between 41 and 50 years old, and the remaining 7.0% were 51 or above. With regards to religion, 289 (67.4%) of respondents were Muslims, 41 (9.6%) Buddhist, 56 (13.1%) Hindus, and 42 (9.8%) were Christians. In terms of ethnicity, the respondents were Malay (55.7%), Indian (14,9%), Chinese (13.1%), and others (16.3%).

In terms of occupational affiliation, the majority of the respondents (60.0%) were self-employed, 21.7% worked in the private sector, 10% the public sector, and 7.7% were students. The monthly income data shows that only 7.1% of respondents earn above RM3000, while the other 92.9% made less than RM3000 per month. Referring to level of education, 69.5% of respondents were secondary school leavers and only 6.5% had graduated from undergraduate programs. Meanwhile, in terms of prison re-entry, 45.25% (194) had been incarcerated between two to five times, 39.2% (168) were first-time prisoners, and 15.6% (67) had been in prison six or more times.

The data in Table 2 show that the mean for overall emotional intelligence and all subscales (emotional perception and expression, emotional facilitation of thinking, emotional understanding and emotional management) were within the medium range. The mean scores for pre-contemplation, contemplation, action, and maintenance striving was 2.88, 3.88, 3.84, and 3.59 respectively, which fall from the medium to higher end of the subscale mean. Meanwhile, the mean of readiness to change was only 8.44, which indicates that the samples were mainly in the contemplation state. Based on the rule of thumb for interpreting the size of correlation coefficient [2], there was a low positive correlation between overall emotional intelligence and readiness to change with a correlation coefficient of 0.33. Emotional intelligence was also found to have a significant low positive correlation with the contemplation, action, and maintenance striving stages with correlation coefficients of 0.41, 0.46, and 3.70 respectively. However, the correlation coefficient between emotional intelligence and pre-contemplation, although significant, was negligible at 0.18.

## 2 Experimental Design, Materials and Methods

Respondents were selected through stratified random sampling by choosing two prisons to represent each of the four zones in Malaysia, namely West Malaysia, East Malaysia, Central Malaysia and Borneo. The researchers randomly administered 550 surveys to the inmates in the prison. Although the response rate was 100%, only 78% (*n* = 429) was used for analyses since 121 surveys have to be dismissed due to missing data and outliers.

Data collection was done using questionnaires to measure the variables involved. To measure emotional intelligence, the researchers used the Self-Rated Malaysian Emotional Intelligence Scale (SRMEIS) from Ida Hartina Ahmed Tharbe and Ng [3], which was developed using the Mayer and Salovey [4] emotional intelligence framework. A total of 39 items represented the four subscales of emotional intelligence, whereby 11 items measured emotional perception and expression, 13 measured emotional facilitation of thinking, 4 items measured emotional understanding, and 11 items measured emotional management. All items of each subscale are as listed in the table. Out of the 39 items, items numbered 3, 6, 10, 20, 30, 37 and 38 are reverse coded. SRMEIS Items were measured by a 5-point Likert scale that ranged from 1 (Strongly Disagree) to 5 (Strongly Agree). Scoring can be done based on each subscale or overall scores. The score interpretation is divided into three levels, namely high emotional intelligence, average emotional intelligence and low emotional intelligence. Table 3 shows the SRMEIS interpretation of scores for independent subscales and overall emotional intelligence. The reliability of SRMEIS was found to have a Cronbach`s Alpha score of 0.86.

**Table 3:** SRMEIS Scores interpretation for overall and subscales of emotional intelligence.

No	Subscales & Items no.	Score Range	Score Interpretation on the level of emotional intelligence
1.	Emotional Perception and Expression (1, 4, 7, 11, 14, 17, 21, 24,27, 31, 34)	11–55	Low 11 to 25 Medium 26 to 40 High 4 to 55
2.	Emotional Facilitation of Thinking (2, 5, 7, 12, 15, 18, 22, 25, 28, 32, 35, 38, 39)	13–65	Low 13 to 30 Medium 31 to 47 High 48 to 65
3.	Emotional Understanding (10, 20, 30, 37)	4–20	Low 4 to 9 Medium 10 to 14 High 15 to 20
4.	Emotional Management (3, 6, 9, 13, 16, 19, 23, 26, 29, 33, 36)	11–55	Low 11 to 25 Medium 26 to 40 High 41–55
5.	Total emotional intelligence score (inclusive of all 39 items)	39–195	Low 39 to 90 Medium 91 to 142 High 143 to 195

The stages of change were measured using URICA or University of Rhodes Island Change Assessment Scale [5], which was translated into the Malay language using the back-translation method. The translated scale was later evaluated for reliability and validity. This research used the 32-item version of URICA that has four subscales, namely pre-contemplation, contemplation, action, and maintenance striving stage. Each subscale has eight items measured by a 5-point Likert scale that ranged from 1 (Strong Disagreement) to 5 (Strong Agreement).

The mean subscale scores of the four factors ranged from 1 to 5. To obtain the readiness to change score, which is the second-order structure, the pre-contemplation mean score was subtracted from the sum of the means from the contemplation, action, and maintenance subscales (Readiness = C + A + M – PC). Higher scores reflect a higher readiness to change, whereas a score of eight and below represents a pre-contemplation state. A score between 8.01 and 11.99 represents a contemplation state, and scores of over 12 represent a preparation state Content validity was confirmed by two experts in the field. Construct validity was tested between the English and Translated versions of the instrument by tests of test-retest. In this current dataset, A Cronbach's alpha score of 0.84 for the change scale indicates the high reliability of the instrument.

Data were analysed using IBM SPSS Statistics Version 25 in terms of descriptive statistics and correlation. All data are provided in the supplementary file.

## Ethics Statement

Ethical approval to conduct the study was obtained from Malaysian Prison Department under the Ministry of Home Affairs Malaysia (KDN 500-7/1/4 JLD2.(85)). Participation of respondents was totally voluntary and informed consent was gathered before collection of data. The collection of data was done according to the principles of research ethics.

## Declaration of Competing Interest

The authors wish to confirm that there are no known conflicts of interest associated with this publication and there has been no significant financial support for this work that could have influenced its outcome.

The authors confirm that the manuscript has been read and approved by all named authors and that there are no other persons who satisfied the criteria for authorship that are not listed. The authors further confirm that the order of authors listed in the manuscript has been approved by each of us.

The authors confirm that due consideration has been given to the protection of intellectual property associated with this work and that there are no impediments to publication, including the timing of publication concerning intellectual property. In doing so, the authors confirm that the regulations of the institutions concerning intellectual property has been followed.
